# Identification and validation for biomarkers associated with mitochondrial metabolism in chronic obstructive pulmonary disease

**DOI:** 10.3389/fmed.2025.1612390

**Published:** 2025-08-25

**Authors:** Wenjun Wang, Lanxiang Wu, Chao Ouyang, Tao Huang

**Affiliations:** ^1^State Key Laboratory of Respiratory Diseases, Guangzhou Medical University, Guangzhou, China; ^2^Department of Neurology, The Second Affiliated Hospital, Jiangxi Medical College, Nanchang University, Nanchang, China; ^3^Department of Respiratory Diseases, Nanchang People's Hospital, Nanchang, China

**Keywords:** chronic obstructive pulmonary disease, drug forecasting, mitochondrial metabolism, Immune infiltration, biomarkers

## Abstract

**Background:**

Chronic obstructive pulmonary disease (COPD) is a chronic respiratory disease. However, the biological role of mitochondrial metabolism (MM) in COPD remains poorly understood. This study aimed to explore the underlying mechanisms of MM in COPD using bioinformatics methods.

**Methods:**

The datasets GSE57148 and GSE8581 were downloaded from Gene Expression Omnibus (GEO), and 1,234 mitochondrial metabolism-related genes (MM-RGs) were downloaded from the literature. In GSE57148 dataset, differentially expressed genes (DEGs) were determined. The intersection of DEGs and MM-RGs was taken to obtain candidate genes. Protein–protein interaction (PPI) network was used to obtained candidate key genes. Machine learning was employed to detect key genes. The biomarkers were identified through expression validation and receiver operating characteristic (ROC) curves. Subsequently, a nomogram was developed to forecast the likelihood of developing COPD. In addition, functional enrichment analysis, immune infiltration, molecular regulatory network, and drug prediction were carried out. Finally, reverse transcription-quantitative polymerase chain reaction (RT-qPCR) and immunohistochemistry analysis were used to verify DEGs of lung tissues of COPD patients and controls.

**Results:**

Adenine phosphoribosyltransferase (APRT) and lecithin-cholesterol acyltransferase (LCAT) were identified as potential biomarkers. Subsequently, a nomogram was formulated based on these two biomarkers, revealing their significant diagnostic potential. Pathways co-enriched by two biomarkers included ribosome, among others. Immune infiltration analysis showed that 15 types of immune cells were differential immune cells. APRT predicted a total of 30 miRNAs and LCAT predicted a total of 17 miRNAs. APRT was predicted to be targeted by 30 microRNAs (miRNAs), while LCAT was associated with 17 miRNAs. Additionally, 178 transcription factors (TFs) were predicted to regulate APRT, and 86 TFs were predicted for LCAT. TFs shared by both biomarkers include SPI1, CTCF and BCL3, etc. Finally, drug prediction analysis found a total of 114 target drugs for APRT and 156 target drugs for LCAT. The mRNA and protein expression of APRT and LCAT were significantly decreased in COPD patients’ lung tissues.

**Conclusion:**

APRT and LCAT were identified as biomarkers for COPD, and this provides deeper understanding into the mechanisms behind COPD and identifies potential markers for early diagnosis and therapeutic intervention.

## 1 Introduction

Chronic obstructive pulmonary disease (COPD), the third leading cause of death globally, is a chronic respiratory disease with incomplete reversible and progressive airflow restriction (1, 2). COPD is not only a serious threat to human health, but also brings a heavy burden to the medical system and social and economic development because of its high incidence, disability rate, and repeated acute exacerbations (1, 2). The main symptoms of COPD are cough, sputum, shortness of breath, chest tightness and decreased activity tolerance, and the critical pathological features were small airway remodeling, emphysema, and airway inflammation (3). Current treatments of COPD include bronchodilators, oxygen therapy, respiratory training and rehabilitation, and surgery, which aims to control symptoms and reduce the frequency and severity of acute exacerbations (4). Although recent clinical and translational studies focusing on COPD, its etiology and pathogenesis were not fully elucidated (5). Therefore, excavating new biomarkers and exploring the molecular mechanism of COPD are of great significance for exploring targeted treatment strategies and improving patient prognosis.

Mitochondria are double-membraned organelles found in most eukaryotic cells and are deeply involved in metabolism, cell growth, and cell death (6). They serve as the primary intracellular sites for aerobic respiration, converting nutrients such as carbohydrates, fats, and amino acids into adenosine triphosphate (ATP) through oxidative phosphorylation, thereby supplying the essential energy currency required to power diverse cellular activities (7). Mitochondrial metabolism (MM) refers to the bioenergetic processes encompassing both energy production and utilization carried out by mitochondria within cells, including reactive oxygen species (ROS) oxidative phosphorylation (OXPHOS), ATP, and so on (8, 9). Furthermore, emerging evidences indicated that MM activities critically modulate cellular redox homeostasis and participate in maintaining intracellular equilibrium, with their functional integrity being mechanistically linked to diseases and aging processes (8, 10). For instance, an investigation focusing on pancreatic ductal adenocarcinoma (PDA) showed that pancreatic cells expressing oncogenic Kras had higher level of 4HNE (4-Hydroxy-2-nonenal), a marker for mitochondrial oxidative stress, and Mito-Q, the mitochondria-targeted antioxidant, could reduce Kras-caused formation of pancreatic abnormal structures in mice (11). Furthermore, disruption of mitochondrial function due to deficiency of the mitochondrial transcription factor A (TFAM) gene could decrease tumorigenesis in an oncogenic Kras-driven mouse model of lung cancer (12). These evidences suggested that MM and mitochondrial ROS generation are critical to tumorigenesis (11, 12).

Recently, increasing attention have been given to the role of MM in the occurrence and development of COPD by scholars. Mitochondrial impairment in airway epithelium was observed in COPD animal models (13). Notably, cigarette smoke extract (CSE)-exposed airway epithelial cells demonstrated downregulation of Sirtuin 3 (Sirt3), a mitochondrial-localized deacetylase critical for mitochondrial functional regulation, accompanied by dose-dependent attenuation of the antioxidant enzyme manganese superoxide dismutase (MnSOD) (13). Moreover, previous studies have indicated that prolonged CSE exposure in airway epithelium upregulate the expression of mitochondrial fission and fusion proteins, dysregulate the OXPHOS system, and enhance mitochondrial oxidative stress responses (14). Moreover， compared to smoker controls, airway epithelial cells from COPD patients exhibited elevated mitochondrial oxidative stress markers (14). These results showed that MM was deeply involved in both the pathogenesis and progression of COPD. However, current evidence remains limited to correlational studies delineating MM involvement in occurrence and development of COPD. Consequently, identifying MM-associated biomarkers in COPD and systematically investigating MM regulatory mechanisms hold critical significance for elucidating the pathogenic mechanisms underlying COPD development, as well as advancing novel therapeutic strategies targeting mitochondrial dysfunction.

**Figure 1:**
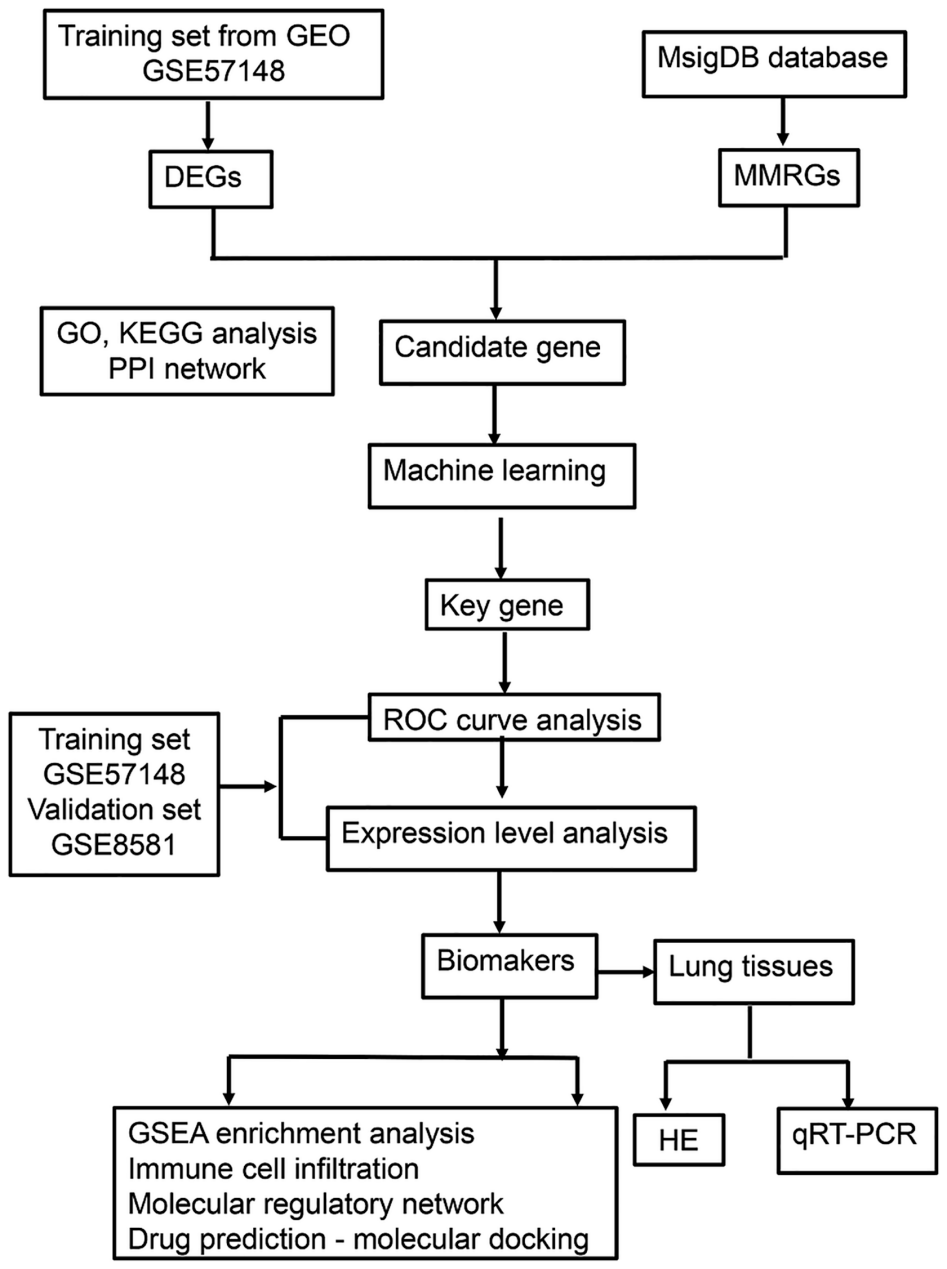
The flowchart of the experiment. GEO, gene expression omnibus; DEGs, differentially expressed genes; MMRGs, mitochondrial metabolism related genes; PPI, protein–protein interaction; ROC, receiver operating characteristic; GSEA, gene set enrichment analysis; HE, hematoxylin–eosin; qRT-PCR, quantitative real-time polymerase chain reaction.

This study (Figure 1) established a multidimensional analytical framework leveraging COPD transcriptomic data from publicly available databases. Machine learning algorithms were employed to identify pivotal genes, followed by integrative analysis of training and validation cohorts to discern COPD-associated biomarkers. Systems biology approaches were subsequently performed to characterize biomarker-enriched biological pathways, delineate immune cell infiltration patterns, construct molecular regulatory networks, and prioritize potential therapeutic agents. Lastly, experimental validation using COPD patient-derived lung tissue specimens was conducted. This investigation provides novel mechanistic insights for COPD diagnosis, evaluation, and management.

## 2 Materials and methods

### 2.1 Data source

The Gene Expression Omnibus (GEO) database^1^ was employed for obtain transcriptomic data from COPD. Specifically, GSE57148 (GPL11154) contained 184 lung tissue samples, including 95 COPD patient’s lung tissue samples and 89 normal lung tissue samples (control samples) (15). In addition, GSE8581 dataset (GPL570) includes 35 lung tissue samples, comprising 16 from COPD patient’s lung tissueand 19 from control lung tissue (16). The database was accessed on December 9. A total of 1,234 mitochondrial metabolism-related genes (MM-RGs) were retrieved from previously published literature (17).

### 2.2 Identification and analysis of candidate genes

Principal component analysis (PCA) was performed on the GSE57148 dataset (COPD vs. control) using the *prcomp* function from the “stats” package (v 4.2.2) (18) to identify and remove outlier samples. The “scatterplot3d” package (v 0.3.42) (19) was used to visualize clustering of different sample groups. Differentially expressed genes (DEGs) were obtained in GSE57148 dataset (COPD vs. control) using the “DESeq2” (v 1.38.0) (20) with *p* < 0.05 and |log2fold change (FC)| > 0.5 as criteria. A volcano plot and a heatmap were generated using the R packages “ggplot2” (v 3.4.3) (21) and the “ComplexHeatmap” (v 2.14.0) (22), respectively, to visualize the top 10 upregulated and downregulated genes, ranked by their log_2_ fold change (log_2_FC) values. The intersection of DEGs and MM-RGs was used to identify candidate genes using the “VennDiagram” (v 1.7.3) (23). Finally, Gene Ontology (GO) and Kyoto Encyclopedia of Genes and Genomes (KEGG) analyses were conducted using the R package “clusterProfiler” (v 4.7.1.003) (24)(*p* < 0.05). The STRING^2^ was utilized for protein–protein interaction (PPI) network construction. The threshold was an interaction score ≥0.4. Results were visualized using the Cytoscape software (v 3.10.2) (25). To find the candidate key genes, a total of 3 algorithms (Stress, Bottleneck, and Betweenness) of CytoHubba plugin in Cytoscape software (v 3.10.2) were used to assess the importance of each candidate gene, and the candidate gene with top 10 of each algorithm were selected and visualized. The intersection of the top 10 candidate genes from three different algorithms were obtained as candidate key genes using the R package “UpSet” (v 1.4.0) (26).

### 2.3 Identification of biomarkers

Machine learning was employed to identify the key genes. First, in GSE57148 dataset, based on candidate key genes, the support vector machine–recursive feature elimination (SVM-RFE) algorithm was constructed using the R package “caret” (v 6.0–93) (27) for svmRadial function to identity SVM-RFE-genes, and a 10-fold cross validation was performed. Second, in GSE57148 dataset, based on candidate key genes, the Boruta algorithm was built using the “Boruta” (v 8.0.0) (28) to identity Boruta genes. Finally, the key genes were identified by intersecting SVM-RFE genes and Boruta genes using R package “ggVennDiagram” (v 1.2.2) (29). The R package “pROC” (v 1.18.0) (30) was employed to plot receiver operating characteristic (ROC) curves in GSE57148 and GSE8581 datasets to assess the capacity of key genes to distinguish between COPD and control samples, with area under curve (AUC) values being computed. The key genes exhibiting an AUC value above 0.70 under the ROC curve in both the GSE57148 and GSE8581 datasets were identified as candidate biomarkers. In addition, Wilcoxon was utilized to compare the levels of expression of candidate biomarkers between COPD and control samples in GSE57148 and GSE8581 datasets (*p* < 0.05). The candidate biomarkers that exhibited consistent expression patterns and statistically significant differences among GSE57148 and GSE8581 datasets were identified as biomarkers. A boxplot was then created using the R package “ggplot2” (v 3.4.3) to show results. A nomogram was developed utilizing the biomarkers in GSE57148, employing the R package “rms” (v 6.5.0) (31). In GSE57148, the calibration curves of the nomogram were employed to assess its predictive accuracy using the “ResourceSelection” (v 0.3.5) (32) (*p* > 0.05). The ROC curve for the nomogram was generated utilizing the “pROC” (v 1.18.0), and the AUC value was used to evaluate its capacity for accurate diagnosis for COPD, with an AUC > 0.7 indicating good performance. The reliability of the nomogram was evaluated through decision curve analysis using the “rmda” (v 1.6) (33).

### 2.4 Functional analysis of biomarkers

To examine the chromosomal localization of biomarkers, the R package “RCircos” (v 1.2.2) (34) was used to visualize their location on chromosomes. GSEA analysis was carried out. First, the Spearman correlation coefficient between each biomarker and other genes was computed using the “psych” (v 2.2.9) (35). The correlations between the biomarkers and other genes were then ranked in descending order determined based on their correlation coefficients. Subsequently, the c2.kegg.v7.4.symbols gene set was acquired from Molecular Signatures Database (MSigDB)^3^ to serve as the background gene sets (v 7.5.1) (36). Finally, the “clusterProfiler” (v 4.7.1.003) (24) was used for GSEA pathway enrichment analysis of biomarker (normalized enrichment score (NES)| > 1, FDR < 0.25, *p* < 0.05). The “enrichplot” (v 1.18.3) (37) was employed to visualize the top 10 results by *p* value.

### 2.5 Immune infiltration

First, the immune abundance of 28 immune cells (38) in GSE57148 between COPD and control samples was determined using the “GSVA” (v 1.46.0) (39) ssGSEA algorithm. The “pheatmap” package (v 1.0.12) (40) was used to visualize the distribution of enrichment scores of 28 immune infiltrating cells. The Wilcoxon test was used to assess differences in immune cell infiltration between the two groups, and the differential immune cells were identified as differential immune cells (*p* < 0.05), with visualizations created using the “ggplot2” (v 3.4.3) to construct boxplot. Subsequently, Spearman correlation analysis was performed using the ‘psych’ package (v2.2.9) to evaluate associations among the differential immune cells, with correlations considered significant at |correlation coefficient (cor)| > 0.3 and *p* < 0.05. Correlation heatmaps were created using the “ggplot2” (v 3.4.3). Spearman correlation analysis was conducted between the differential immune cells and the identified biomarkers under the same thresholds (|cor| > 0.3 and *p* < 0.05), and the results were also visualized using correlation heatmaps in “ggplot2”.

### 2.6 Establishment of the network

The miRNAs associated with biomarkers were predicted using miRWalk^4^ and mirDIP.^5^ The intersecting miRNAs from both databases were identified using the “VennDiagram” package (v 1.7.3). The Cytoscape software (v 3.10.2) was utilized for visualization purposes. Transcriptional regulation is a crucial aspect of gene expression control, wherein transcription factors (TFs) exercise their regulatory function by binding to specific nucleotide sequences located upstream of a gene’s promoter region. The hTFtarget database^6^ was used to predict transcription factors (TFs) associated with the biomarkers, and a graphical network representation of TF–mRNA interactions was constructed using Cytoscape software (v 3.10.2).

### 2.7 Drug prediction and molecular docking

To identify potential therapeutic drugs associated with biomarkers, the Comparative Toxicogenomics Database^7^ was employed to forecast potential medications that interact with the biomarkers. Drugs were ranked from the highest to the lowest interaction score, and the top 5 drugs with the highest interaction scores for each biomarker were displayed. Molecular docking was performed using the top-scoring drugs and their corresponding biomarkers. First, the 3D structures of the target proteins (biomarkers) were downloaded from AlphaFold.^8^ Next, the 3D structures of the molecular ligands (key active ingredients) were obtained from PubChem.^9^ Docking simulations were then carried out using the CB-Dock2 platform.^10^

### 2.8 Subjects

According to the diagnostic criteria of the 2023 revision of the Global Initiative for Chronic Obstructive Pulmonary Disease (GOLD), the patients were divided into COPD group and normal lung function control group. Selection criteria were as follows: patients who underwent lobectomy due to pulmonary nodules; pulmonary function index tests and mMRC (modified British medical research council) tests were performed preoperatively; patients could provide detailed medical history, such as smoking history and history of comorbidities. The pathological examination of the resection margin confirmed the absence of concurrent tumors. The exclusion criteria were as follows: age under 18 years old; presence of chronic respiratory diseases such as asthma, bronchiectasis, interstitial lung disease; presence of chronic infectious diseases, such as chronic viral hepatitis, tuberculosis; undergoing chemotherapy, radiotherapy, theophylline, anticholinergic drugs, adrenal cortex hormone, catecholamine, and beta blockers, which could affect lung function in 3 months; presence of an autoimmune system disease, such as systemic lupus erythematosus (SLE) and rheumatoid arthritis; presence of other systemic chronic uncontrolled diseases such as hypertension, diabetes, coronary heart disease, severe liver and kidney impairment, neurological disorders and so on.

Pulmonary tissue specimens were obtained through post-surgical collection following lung resection procedures from five individuals diagnosed with COPD and five normal lung function individuals as controls at the Second Affiliated Hospital of Nanchang University. One portion of the lung tissue was fixed in 4% paraformaldehyde for paraffin-embedding, while the other portion was snap-frozen in liquid nitrogen for subsequent RNA extraction. Subject characteristics were presented in Table 1. The study was performed in accordance with the Declaration of Helsinki, and was approved by the Ethics Committee of the Second Affiliated Hospital of Nanchang University. All patients provided written informed consent.

**Table 1:** Subject characteristics.

Item	Control group	COPD group
Total of subjects (*n*)	5	5
Sex [male, *n* (%)]	4 (80)	4 (80)
Age (year, mean ± SD)	40.55 ± 9.70	47.36 ± 10.52
Smoking index (pack years, mean ± SD)	27.63 ± 15.38	35.73 ± 16.70
Disease constitution carcinoma, *n* (%)	3 (75)	4 (80)
FEV1 (L, mean ± SD)	2.37 ± 0.89	1.81 ± 0.56*
FVC (L, mean ± SD)	2.99 ± 0.74	3.11 ± 0.63
FVC% pred (mean ± SD)	82.74 ± 7.26	79.25 ± 6.31
FEV1/FVC (mean ± SD)	79.21 ± 8.78	60.23 ± 9.14*
FEV1%pred (mean ± SD)	90.42 ± 11.77	63.59 ± 14.43*
Frequency of AE (times/year, mean ± SD)	1.58 ± 0.72*	0
mMRC (mean ± SD)	0	1.40 ± 0.55*

COPD, chronic obstructive pulmonary disease; FEV1, forced expiratory volume in the first second; FVC, forced vital capacity; AE, acute exacerbation. mMRC, modified british medical research council. *p < 0.05 vs. control group.

### 2.9 Histological staining, reverse transcription-quantitative polymerase chain reaction (RT-qPCR) and immunohistochemistry analysis

Hematoxylin–eosin (HE) staining was used to observe the morphological changes of lung tissues. Transparent tissue sections were prepared from wax blocks using a paraffin microtome (RM2235, Leica, Wetzlar, Germany). And, these sections were dewaxed, hydrated, and stained with HE. Finally, the stained samples were observed under an optical microscope (DP80, Olympus, Tokyo, Japan). The images were analyzed under 200-fold magnification, and three visual fields were randomly selected for analysis per sample. Five alveoli cross-sectional areas (CSA) in one field were measured to calculate the average CSA of this field. The average of the three fields was considered as the CSA of this specimen.

RT-qPCR was utilized for confirm the expression levels of the biomarkers of lung tissues. Extraction of total RNA was carried out from 10 samples using Trizol (Ambion, Austin, USA), adhering strictly to the manufacturer’s instructions. The total RNA was transcribed into cDNA utilizing the SurescriptTM First-strand cDNA Synthesis Kit (Servicebio, Wuhan, China), in accordance with the manufacturer’s protocol. QPCR was carried out with the 2X Universal Blue SYBR Green qPCR Master Mix (Servicebio, Wuhan, China). The relative expression of mRNA was calculated by 2 − △△CT. Primer sequences for adenine phosphoribosyltransferase (APRT) were F: 5′-ATCGACTACATCGCAGGTCTG-3; R: 5′-GCCTTCCCATACTCTAGAGAATAG-3′. Primer sequences for and lecithin-cholesterol acyltransferase (LCAT) were F: 5′-ACCTGGTCAACAATGGCTACG-3′; R: 5′-ACCTGGTCAACAATGGCTACG-3′. Primer sequences for GAPDH were F: 5′-CAAGGTCATCCATGACAACTTTG-3′; R: 5′-GTCCACCACCCTGTTGCTGTAG-3′. Finally, the PCR results were imported into GraphPad for statistical analysisand plotting.

Transparent tissue sections were prepared from wax blocks, and dewaxed, hydrated, blocked. Primary antibodies against APRT (abcam ab196558, 1:200) and LCAT (abcam ab109417, 1:200) were applied overnight at 4°C, with species-matched secondary antibodies and 3,3’-Diaminobenzidine Tetrahydrochloride (DAB) chromogen detection. Slides were counterstained with hematoxylin, dehydrated, and mounted. Protein expression was semi-quantitatively analyzed using ImageJ software.

### 2.10 Statistical analysis

Statistical analyses were conducted using R software (v 4.2.2), with the Wilcoxon employed to assess differences among groups. For CSA, RT-qPCR and immunohistochemistry analysis comparisons, the *t*-test was used. *p* value less than 0.05 were considered as significant.

## 3 Results

### 3.1 Identification and enrichment of candidate genes

**Figure 2:**
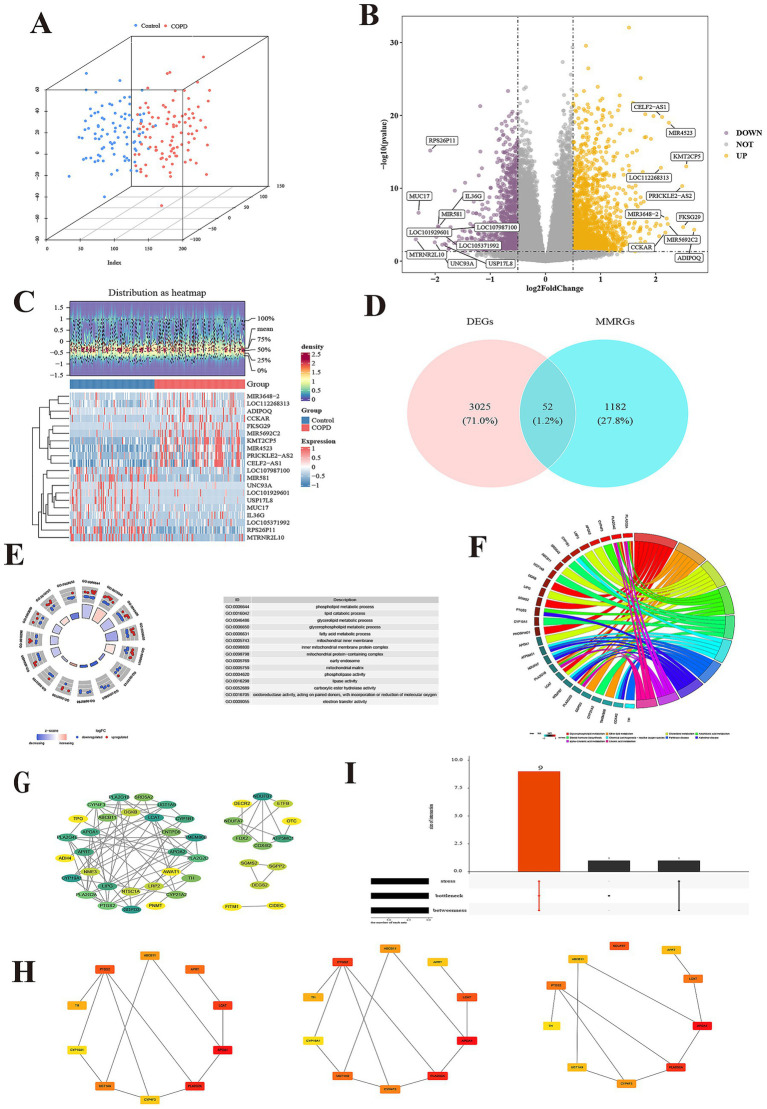
Identification and enrichment of candidate genes. **(A)** The principal component analysis of two group. **(B)** The volcano map of differentially expressed genes (DEGs). **(C)** The heatmap of DEGs. **(D)** Venn diagram of DEGs and mitochondrial metabolism related genes (MMRGs). **(E)** Candidate gene GO enrichment cycle. Each GO item has a corresponding circle, and the color inside the circle represents the logFC value of that entry, with red representing up-regulated expression and blue representing down-regulated expression. **(F)** Enrichment chords of KEGG pathways for candidate genes. Each line represents a different molecular mechanism, has its own unique color, and is marked with a label on the circumference. **(G)** Identification of protein–protein interaction (PPI) networks of candidate genes. The Degree of the protein encoded by the gene ranges from small to large, corresponding to the color from yellow to green. **(H)** Each of the three algorithms scored the top 10 candidate genes, including stress, bottleneck and betweenness. **(I)** Intersection gene of 3 algorithms.

The PCA results show that the COPD and control groups sample had good clustering and no outlier samples (Figure 2A). A total of 3,077 DEGs were determined in GSE57148, including 1,967 up regulated and 1,110 down regulated genes (Figures 2B,C). The DEGs and MM-RGs were selected to be intersected, and 52 candidate genes were detected (Figure 2D). A total of 583 Gene Ontology (GO) biological functions were determined, including 453 biological processes (BP), 26 cellular components (CC), and 104 molecular functions (MF) (*p* < 0.05) (Figure 2E; Supplementary Table 2). In BP, these candidate genes were primarily associated with phospholipid metabolic process, lipid catabolic process, glycerolipid metabolic process, glycerophospholipid metabolic process, and fatty acid metabolic process; In CC, these candidate genes were mainly involved in mitochondrial inner membrane, inner mitochondrial membrane protein complex, mitochondrial protein-containing complex, early endosome and mitochondrial matrix. In MF, these candidate genes were mainly involved in phospholipase activity, lipase activity, carboxylic ester hydrolase activity, oxidoreductase activity acting on paired donors, with incorporation or reduction of molecular oxygen and electron transfer activity. KEGG identified a total of 36 enriched functional pathways, specifically: glycerophospholipid metabolism, ether lipid metabolism, cholesterol metabolism, arachidonic acid metabolism, alpha-Linolenic acid metabolism, linoleic acid metabolism, steroid hormone biosynthesis, chemical carcinogenesis - reactive oxygen species, Parkinson disease, and Alzheimer disease (*p* < 0.05) (Figure 2F; Supplementary Table 3). The PPI network was built utilizing the candidate genes, comprising 74 interactions among 42 proteins, and there were 10 proteins that do not interact with other proteins. Notably, the TMEM86B, APOA2, and GDPD3 protein interacts with most of the proteins in the network (Interaction score ≥ 0.4) (Figure 2G). The top 10 candidate genes for each algorithm were obtained using three algorithms from the cytoHubba plugin (Figure 2H), through the overlap of derived from various algorithms, and a total of 9 key genes were identified (Figure 2I; Supplementary Tables 4–6).

### 3.2 APRT and LCAT as biomarkers

**Figure 3:**
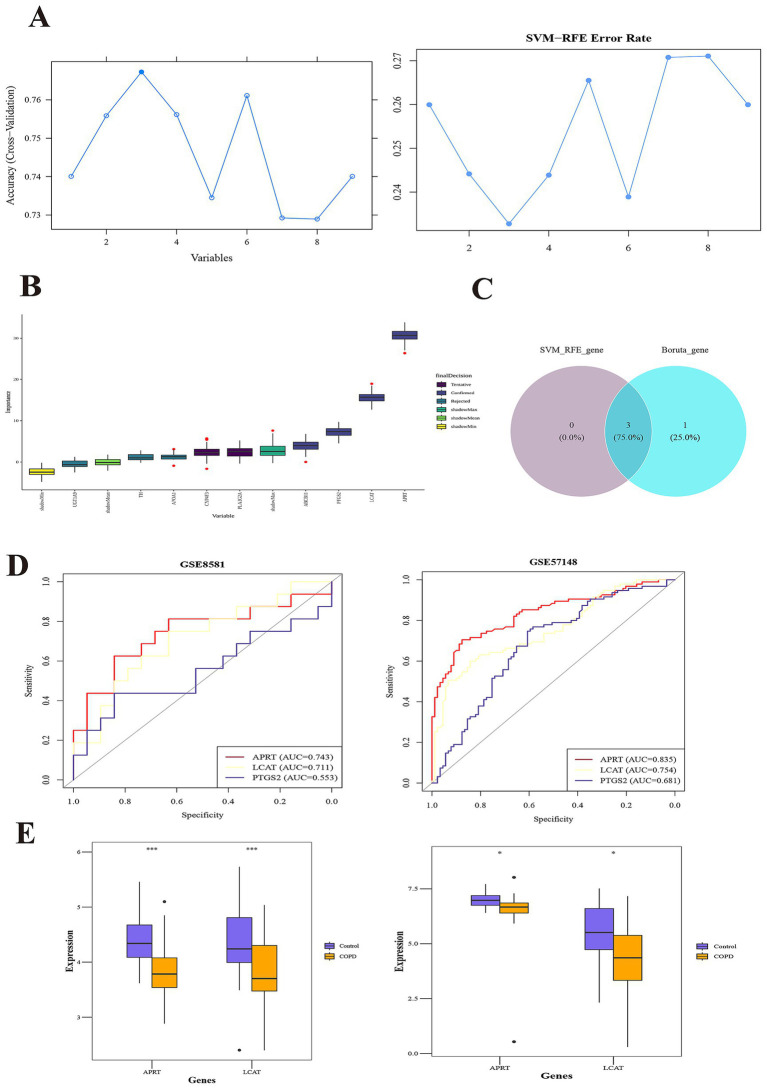
APRT and LCAT as biomarkers. **(A)** SVM-RFE (Support vector machine recursive feature elimination) analysis identified 3 genes. The abscissa indicates the number of feature genes, and the ordinate represents the cross-validated accuracy. **(B)** The Boruta algorithm identified 4 Boruta-genes. The yellow box plots depict the minimum values of shaded attributes, lime green denotes their mean values, and dark green corresponds to the maximum values. Purple boxes indicate rejected genes, while blue boxes signify confirmed genes. **(C)** The overlapping SVM-RFE-feature genes and Boruta-feature genes. **(D)** Receiver Operating Characteristic (ROC) curves of key genes in the training set (left) and validation set (right). The abscissa denotes the False Positive Rate (FPR; 1 – Specificity), and the ordinate represents the True Positive Rate (TPR; Sensitivity). The Area Under the Curve (AUC) quantifies the diagnostic performance of the ROC analysis. **(E)** Box plots of expression of candidate biomarkers in the training set (left) and validation set (right).

A total of 3 SVM-RFE-genes (APRT, LCAT, and PTGS2) were acquired from GSE57148 dataset (Figure 2A). The Boruta algorithm identified 4 Boruta genes from GSE57148—PTGS2, LCAT, ABCB11, and APRT (Figure 3B). Then, by overlapping SVM-RFE-feature genes and Boruta-feature genes, APRT, LCAT, and PTGS2 were identified as key genes (Figure 3C). Subsequently, ROC curves were plotted for these three key genes in both GSE57148 and GSE8581 datasets, the objective of plotting these curves was to evaluate their capacity to differentiate between COPD and control samples. Notably, the AUC values for APRT and LCAT exceeded 0.7 in GSE57148 and GSE8581 datasets; therefore, a total of two candidate biomarkers were obtained (Figure 3D). In the GSE57148 and GSE8581 datasets, validation of the expression levels for the two candidate biomarkers was carried out. The findings indicated that both candidate biomarkers exhibited notably decreased expression in the COPD group, with consistent expression trends observed across both datasets (*p* < 0.05). Therefore, the APRT and LCAT were selected as biomarkers for this study (Figure 3E).

### 3.3 Constructing a model with exceptional diagnostic capability for COPD

**Figure 4:**
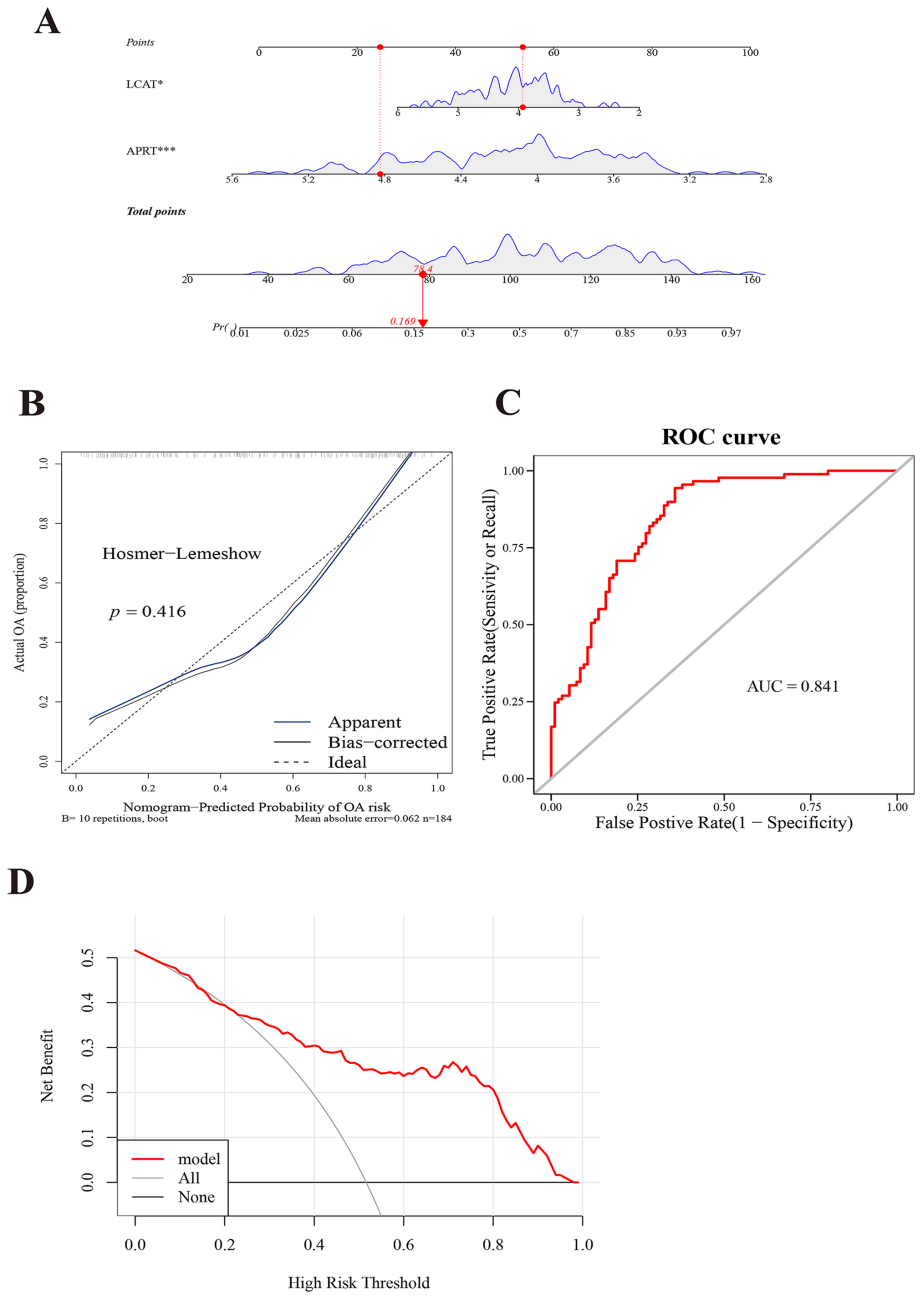
Constructing a model with exceptional diagnostic capability for COPD. **(A)** A nomogram of APRT and LCAT. The abscissa for points/total points corresponds to scoring metrics, whereas the abscissa for the two biomarkers indicates quantitative gene expression values. **(B)** Calibration curve of two biomarkers. The abscissa represents the nomogram-predicted probability of disease, while the ordinate corresponds to the observed probability of disease. **(C)** Receiver Operating Characteristic (ROC) curve analysis of two biomarkers. The abscissa denotes the False Positive Rate (FPR; 1 – Specificity), and the ordinate represents the True Positive Rate (TPR; Sensitivity). The Area Under the Curve (AUC) quantifies the diagnostic performance of the ROC analysis. **(D)** Decision Curve Analysis (DCA) of two biomarkers. The abscissa represents the risk threshold (Pt), and the ordinate corresponds to the net benefit (NB), calculated as benefits minus harms. The curves in the figure illustrate the net benefit across varying risk thresholds.

Subsequently, in the GSE57148 dataset, these 2 biomarkers were integrated into a nomogram to harness their combined diagnostic strengths for COPD. Higher total scores in the nomogram indicated an increased risk of COPD development (Figure 4A). The good consistency of the calibration curve to the ideal curve indicated that the nomogram was more accurate (*p* = 0.416) (Figure 4B). In ROC curve, the AUC value was 0.841; thus, the ROC curve indicated that the nomogram performed well clinical utility (Figure 4C). The DCA curve shows that the nomogram (model) demonstrated markedly elevated performance compared to the diagonal line (All) and the horizontal line (None). These results showed that the nomogram demonstrated favorable clinical net benefits, suggesting its strong clinical applicability (Figure 4D).

### 3.4 Chromosomal localization and GSEA for biomarkers

**Figure 5:**
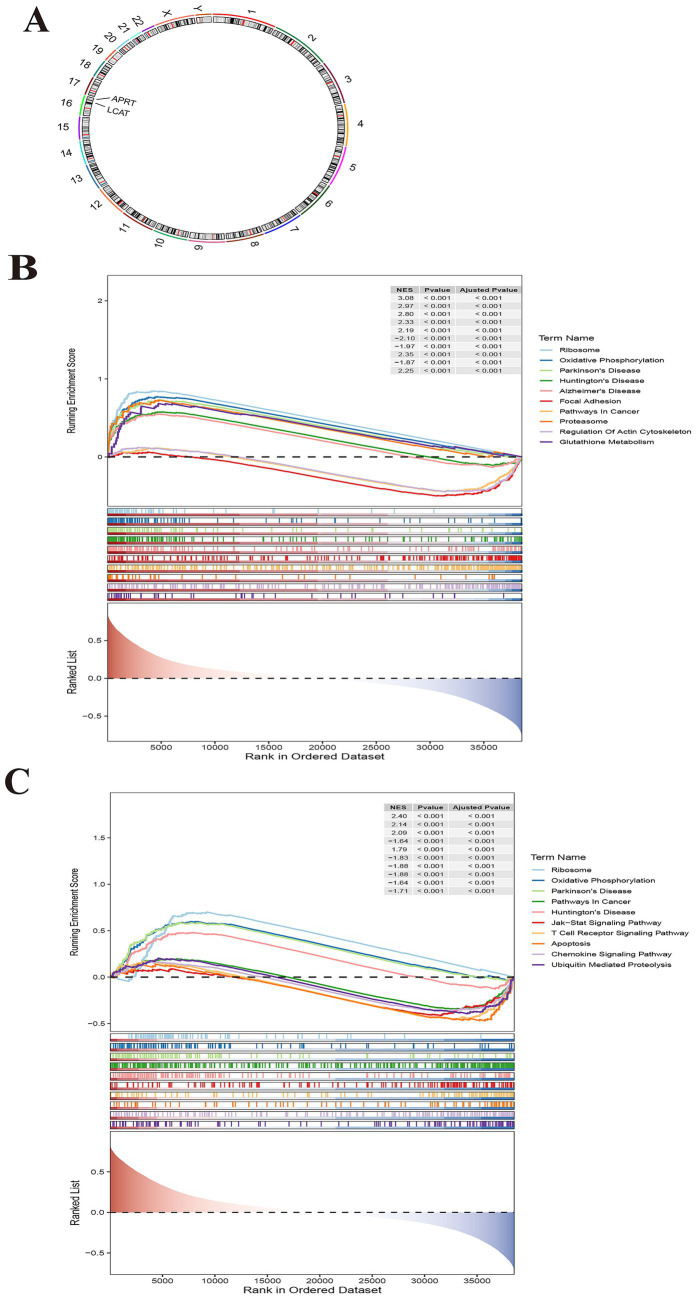
Chromosomal localization and Gene Set Enrichment Analysis (GSEA) for biomarkers. **(A)** Chromosome localization analysis of two biomarkers. APRT and LCAT are localized to chromosome 16. **(B)** GSEA enrichment analysis of biomarker APRT. **(C)** GSEA enrichment analysis of LCAT.

Chromosome localization analysis of biomarkers showed that APRT and LCAT were all on chromosome 16 (Figure 5A). The GSEA results showed that APRT enriched 97 functional pathways, among which the top 10 results were ribosome, oxidative phosphorylation, Parkinson’s disease, Huntington’s disease, Alzheimer’s disease, focal adhesion, pathways in cancer, proteasome, regulation of actin cytoskeleton and glutathione metabolism (Figure 5B; Supplementary Table 7). LCAT was enriched for a total of 74 functional pathways, of which the top 10 results were ribosome, oxidative phosphorylation, Parkinson’s disease, pathways in cancer, Huntington’s disease, Jak–STAT signaling pathway, T cell receptor signaling pathway, apoptosis, chemokine signaling pathway and ubiquitin mediated proteolysis (*p* < 0.05) (Figure 5C; Supplementary Table 8). Understanding the enrichment of LCAT in these pathways can provide insights into its potential roles and interactions within the cell.

### 3.5 A total of 15 differential immune cells

**Figure 6:**
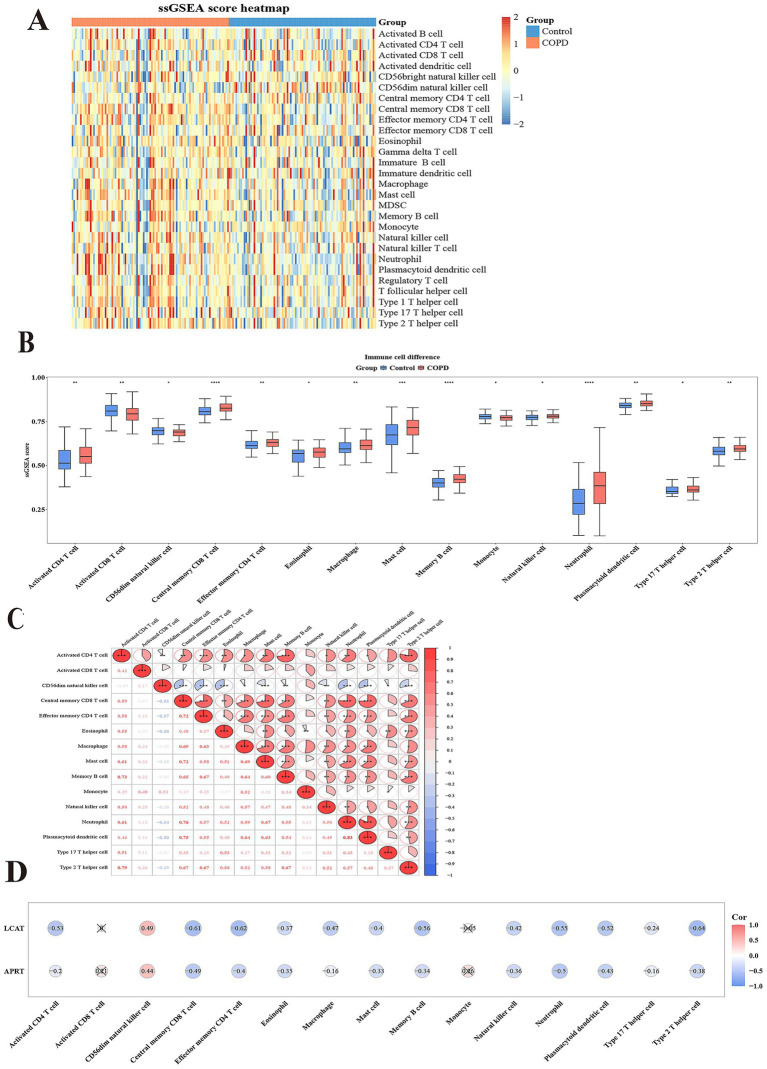
Immunoinfiltration analysis. **(A)** Heatmap of Single sample Gene Enrichment Analysis (ssGSEA). **(B)** 15 types of immunoinfiltrating cells based on the enrichment fraction box plot between COPD group and control group. The abscissa denotes the 15 differential immune cell types, and the ordinate represents their corresponding enrichment scores. **(C)** Heatmap of correlation between 15 different immune cells. Both the abscissa and ordinate represent the 15 differential immune cell types. The intensity of the red hue corresponds to the strength of positive correlations, while the saturation of the blue shade reflects the magnitude of negative correlation coefficients. **(D)** Heatmap of correlation between 15 different immune cells and two biomarkers. The abscissa represents the 15 differential immune cell types, and the ordinate corresponds to the two biomarkers. The intensity of red coloration correlates with stronger positive associations, whereas deeper blue hues indicate larger magnitudes of negative correlation coefficients.

Immune cell infiltration analysis was performed in GSE57148 to obtain 28 immune cells infiltration score between two groups (Figure 6A). A comparison was subsequently conducted to examine the differences in immune cell infiltration between the two groups, and the 15 immune cells were used as differential immune cells (*p* < 0.05) including activated CD4 T cell, etc. (Figure 6B). The correlation analysis between differential immune cells and differential immune cells unveiled a remarkable positive association between plasmacytoid dendritic cell and neutrophil (cor = 0.83, *p* < 0.001), and the strongest negative correlation was effector memory CD4 T cell and CD56dim natural killer cell (cor = − 0.37, *p* < 0.001) (Figure 6C; Supplementary Table 9). The correlation analysis between differential immune cells and biomarkers were a remarkable positive association between LCAT and CD56dim natural killer cell (cor = 0.49, *p* < 0.05); the strongest positive correlation was between APRT and CD56dim natural killer cell (cor = 0.44, *p* < 0.05). However, the strongest negative correlation was between LCAT and Type 2 T helper cell (cor = − 0.64, *p* < 0.05), and the most significant negative correlation was between APRT and Central memory CD8 T cell (cor = − 0.49, *p* < 0.05) (Figure 6D). These correlations provide initial insights into the potential interactions between LCAT, APRT, and specific immune cell subsets. Functional studies could be performed to determine whether LCAT and APRT directly affect the proliferation, differentiation, or function of these immune cells.

### 3.6 Regulatory networks for biomarkers

**Figure 7:**
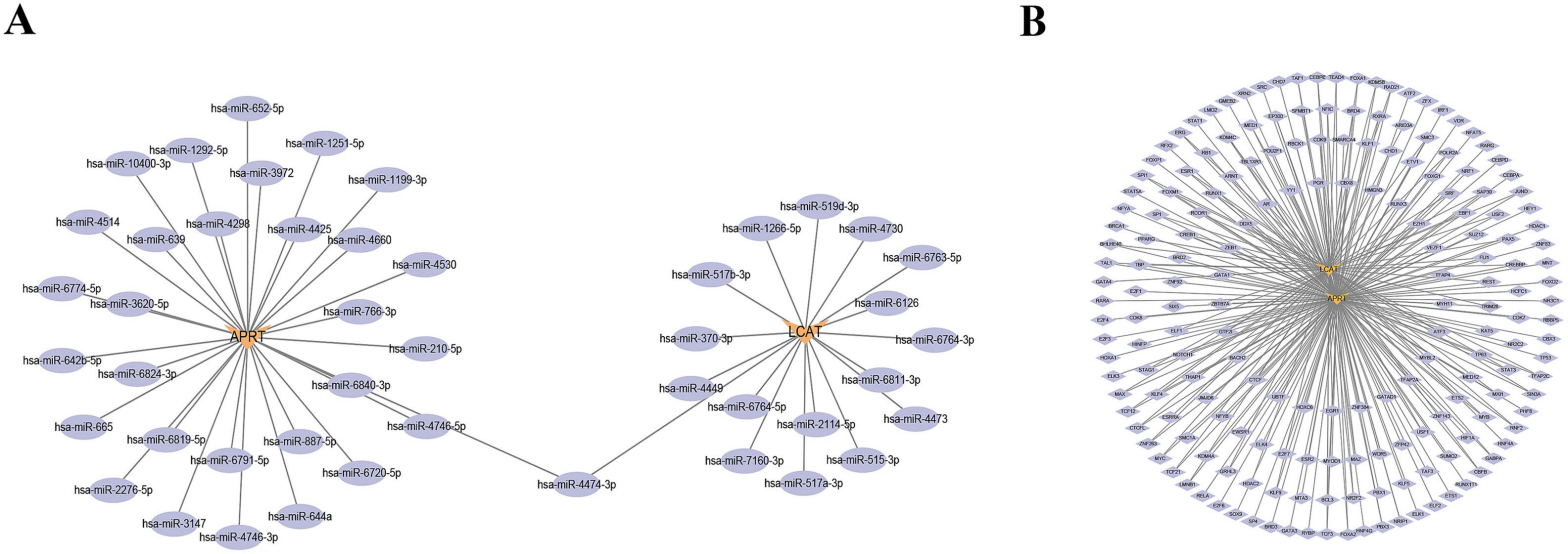
Regulatory networks for biomarkers. **(A)** miRNA-mRNA regulatory network. Orange denotes biomarkers, while purple represents miRNA. **(B)** Transcription Factors (TFs)-mRNA regulatory network. Orange denotes biomarkers, while purple represents TFs.

Firstly, in the miRNA–mRNA network, there were two biomarkers and 47 miRNAs. A total of 30 miRNA were predicted to be regulated by APRT, and a total of 17 miRNA were predicted to be regulated by LCAT; the miRNAs shared by two biomarkers were hsa-miR-4474-3p (Figure 7A; Supplementary Table 10). In addition, the TF-mRNA network was constructed. APRT and LCAT obtained 178 TFs and 86 TFs of acting on biomarkers, respectively. Notably, SPI1, CTCF, and BCL3 were co-regulated by APRT and LCAT, etc. (Figure 7B; Supplementary Table 11).

### 3.7 A total of 270 potential drugs

A total of 270 target drugs were identified for the two biomarkers through database screening. Both biomarkers were predicted to interact with 47 common drugs, including bisphenol A, tetrachlorodibenzodioxin, perfluorooctane sulfonic acid, and sodium arsenite, among others (Supplementary Tables 12, 13).

**Figure 8:**
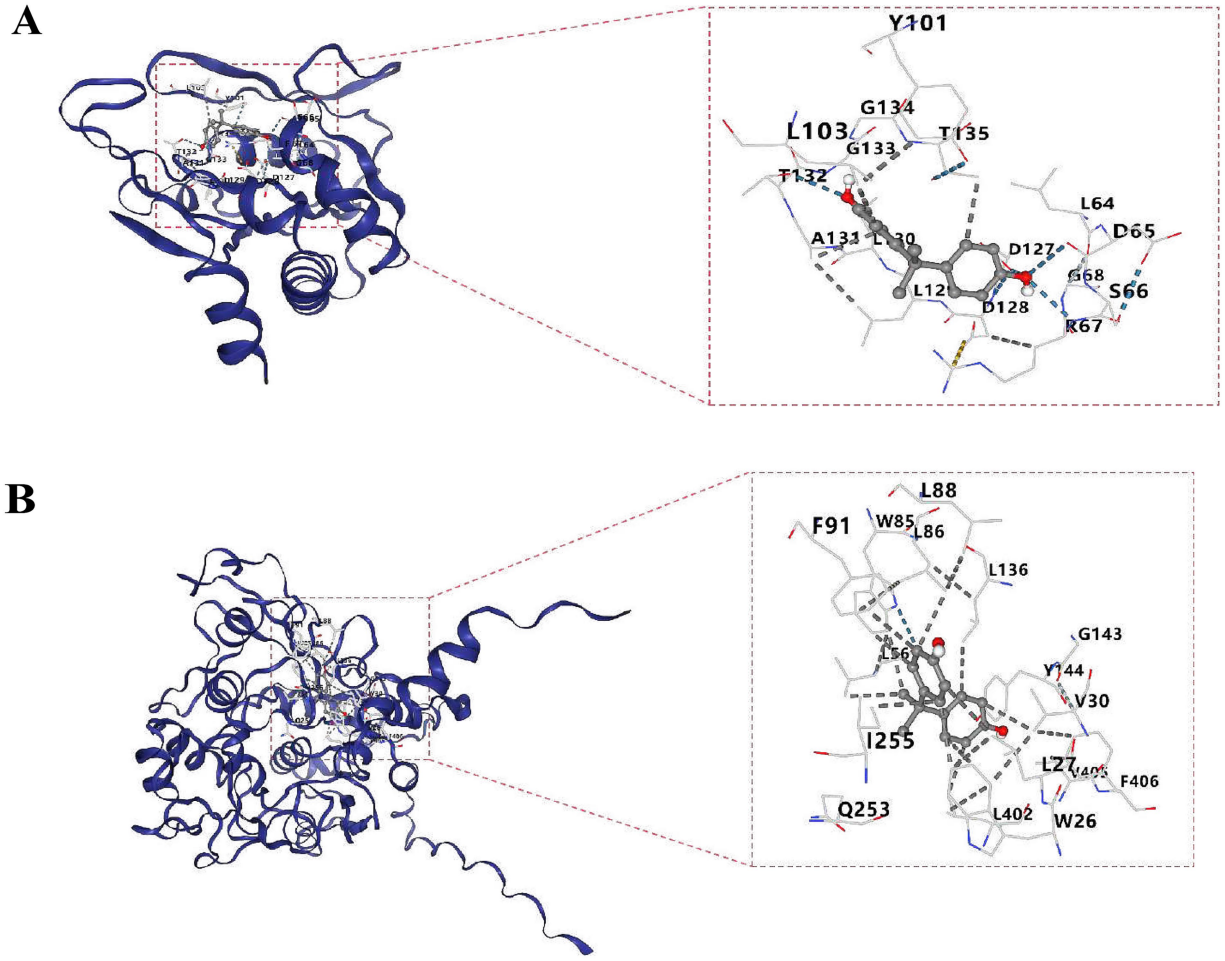
Molecular docking conformation of APRT **(A)** and LCAT **(B)**. The molecular docking of APRT with bisphenol A and LCAT with bisphenol A is shown from top to bottom. The left picture is the whole and the right picture is the partial display. The outermost is the protein skeleton, the middle is the ligand (i.e., drug), and the numbers represent the binding targets.

The molecular docking results showed that the bisphenol A was selected and two biomarkers for molecular docking analysis, respectively. The binding energy between APRT and bisphenol A was −6.6 kcal/mol, while the binding energy between LCAT and bisphenol A was −7.2 kcal/mol (Supplementary Table 14). Molecular docking conformation of APRT interaction with bisphenol A is shown in Figure 8A. Molecular docking conformation of LCAT interaction with bisphenol A was display in Figure 8B.

### 3.8 The verification of APRT and LCAT in lung tissues

**Figure 9:**
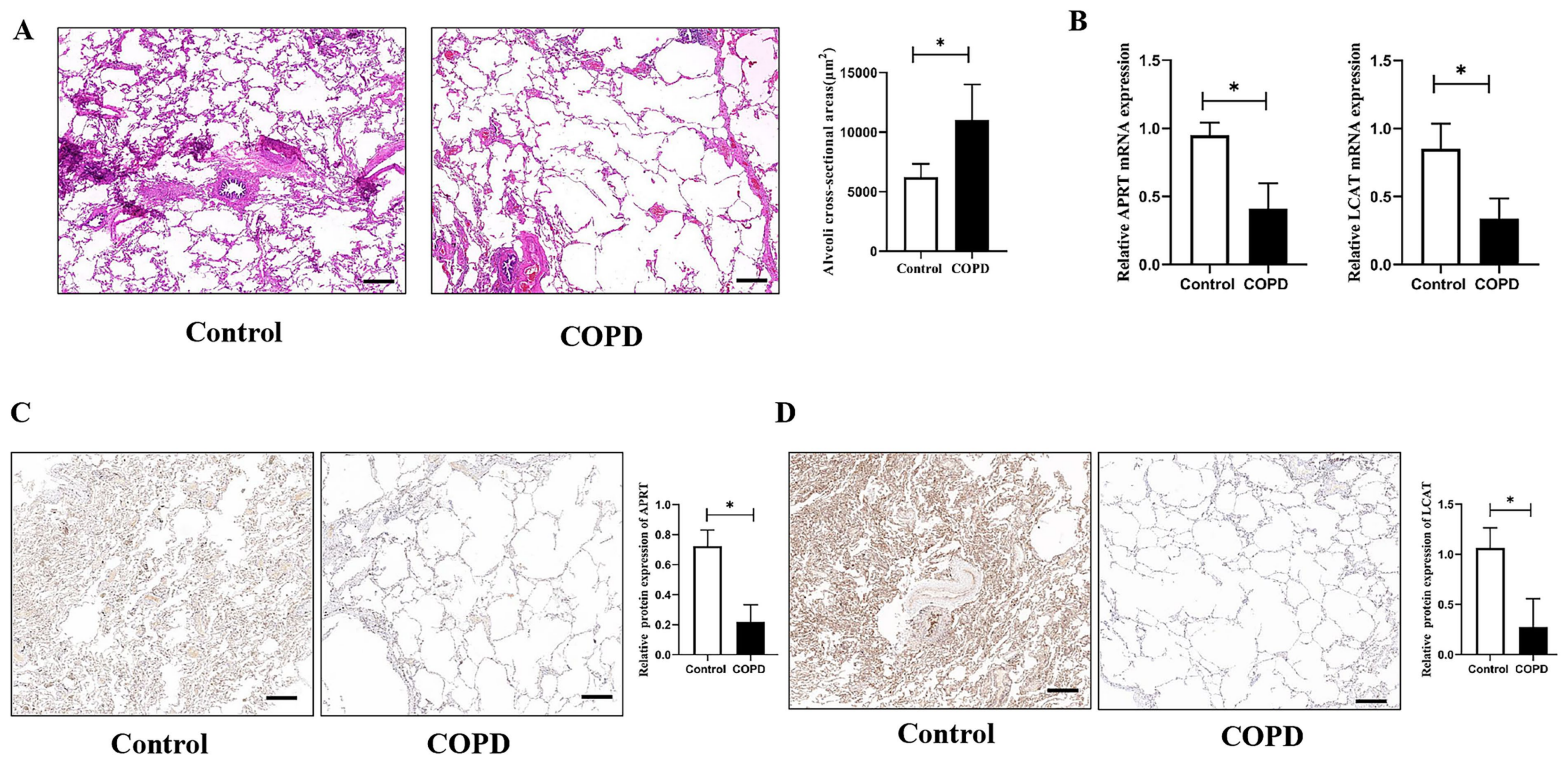
The verification of APRT and LCAT in lung tissues. **(A)** Histological structure of lung tissues. Alveoli cross-sectional areas (CSA) were measured to present emphysema changes in different groups. **(B)** Relative APRT and LCAT mRNA expression between the two groups. **(C)** Immunohistochemistry analysis of the APRT protein expression in lung tissues. **(D)** Immunohistochemistry analysis of the LCAT protein expression in lung tissues. The black scale represents 500 μm. COPD, Chronic obstructive pulmonary disease. *p < 0.05 vs. control group.

As presented in Table 1, there were no significant difference between the two groups regarding to sex, age, smoking index, and composition of diseases. Compared to the control group, lung function index in the COPD group was significantly declined (*p* < 0.05). Also, typical emphysema changes were found in the lung tissues of COPD patients and the alveolar CSA in the COPD patients was significantly larger than the control patients (*p* < 0.05, Figure 9A). The mRNA expression of APRT and LCAT was significantly decreased in COPD lung tissue (*p* < 0.05, Figure 9B). The protein expression of APRT and LCAT was also significantly decreased in COPD lung tissues (*p* < 0.05, Figures 9C,D).

## 4 Discussion

COPD is a common and frequently occurring disease of the respiratory system, with both its incidence and mortality rates exhibiting a steady annual increase worldwide (1, 4). The pathogenesis of COPD involves multiple factors that act in concert, underpinning the complexity of the disease (3). Mitochondrial metabolism serves as the central hub for cellular energy supply and metabolic transformation, and it has been implicated in a wide array of human diseases (41, 42). For instance, the pathological mechanisms of COPD were attributed to aberrant mitochondrial characteristics and mitochondrial dysfunction (43, 44). However, the specific mechanistic role of MM in the pathogenesis of COPD remains to be fully elucidated.

Therefore, this study systematically identified and validated MM-related key genes in COPD through public databases and clinical lung tissue specimens. First, differential expression analysis of the two groups in the training set revealed 3,077 DEGs. Subsequent intersection with MM-RGs yielded 52 candidate genes, which were further explored for their functional annotations and signaling pathways. Next, nine hub genes were identified from these 52 candidates via PPI network analysis, combined with machine learning algorithms (SVM-RFE and Boruta). By integrating data from the training and validation sets, ROC curve analysis and expression validation pinpointed two biomarkers, APRT and LCAT. A diagnostic nomogram based on these biomarkers demonstrated robust predictive performance. Further analyses included chromosomal localization, GSEA, to uncover enriched pathways, differential expression levels in immune cell populations, and the construction of miRNA-mRNA and TF-mRNA regulatory networks at the molecular level. Additionally, potential targeted drugs were predicted and validated through molecular docking. These findings provide a theoretical foundation for future clinical investigations into COPD pathogenesis and therapeutic strategies.

APRT is a pivotal enzyme in the purine salvage pathway, catalyzing the recycling of adenine moieties derived from cellular metabolic byproducts such as polyamine catabolism (45). Its coding sequence (CDS) encodes a 180-amino acid protein that mediates the phosphoribosylation of adenine to form adenosine monophosphate (AMP), a critical step in nucleotide homeostasis (45, 46). APRT was involved in cellular metabolism and energy production, particularly in cell populations requiring rapid nucleotide biosynthesis to sustain DNA and RNA synthesis demands (47, 48). In recent years, the research on APRT has mainly focused on the relationship between APRT deficiency and kidney disease (47, 49–51). For instance, a large pediatric cohort study aiming to assess the clinical presentation, diagnosis, and outcome of APRT deficiency in French reference laboratories, demonstrated that half of the children had decreased kidney function, and two patients presented with acute renal failure (49). Another experiment on chronic intermittent hypoxia (CIH)-induced renal injury showed that APRT overexpression reduced fibrosis and apoptosis, inhibited oxidative stress, and enhanced autophagy in CIH-induced renal tubular epithelial cells, and attenuated the serum levels of blood urea nitrogen in CIH rats (50). These results presented the important role of APRT in kidney metabolism. In addition, a study about diabetic wound healing showed that APRT could utilize adenine to recover cellular proliferation and ATP levels from hydrogen peroxide-induced oxidative damage. Which presented its role during the healing of diabetic wounds (51). Although there were no studies on APRT and COPD, previous studies suggested an important role for APRT in metabolism. In this study, for the first time, the biomarker identity of APRT in mitochondrial bioenergetics of COPD was screened by bioinformatics analysis and verified by lung tissues of COPD patients.

LCAT is a plasma enzyme that catalyzes the esterification of free cholesterol with fatty acids derived from phosphatidylcholine (lecithin), generating cholesteryl esters (52, 53). This biochemical process was essential for maintaining cholesterol homeostasis in plasma and preventing pathological accumulation of cholesterol within vascular walls, thereby serving as a critical mechanism in the prevention of atherosclerosis (52, 54, 55). Current researches were predominantly focused on elucidating the role of LCAT in the pathogenesis and therapeutic modulation of cardiovascular diseases (55, 56). For example，there were investigators who explored the function of LCAT independent of low-density lipoprotein (LDL) clearance effects though a double knockout (LCAT−/−& LDLR−/−, DKO) hamster model and indicated that DKO hamsters presented increased atherosclerotic lesions in the aorta, aortic root, and coronary artery compared to low-density lipoprotein receptor (LDLR)-deficient (LDLR−/−, LKO) hamsters (55). Another study detected the plasma LCAT mass concentration and plaque burden in 267 patients with angiographically proven coronary artery disease (CAD) and 97 control without CAD and found that plasma LCAT mass concentration was increased in CAD patients and negatively related to plaque volume (57). These studies suggested the helpful role of LCAT in suppressing the development of atherosclerotic. Therefore, a plenty of studies and clinical investigations were aimed at promoting plasma lipid metabolism by enhancing the activity of LCAT to find better managements choices for familial LCAT deficiency (FLD) and cardiovascular disease (58). Although no studies to date have investigated the role of LCAT in pulmonary diseases, emerging evidence from research on chronic kidney disease (CKD) offered preliminary insights into its potential systemic regulatory functions (59). In this study, a higher ROS production in renal cells was observed in patients with lower serum LCAT concentration, and decreased plasma LCAT concentration was positively related to CKD development over time in patients with renal dysfunction (59). Downregulation of LCAT mRNA expression was detected in collected COPD lung tissues, indicating its potential involvement in COPD-associated mitochondrial bioenergetics dysregulation, which may contribute to the pathogenesis and progression of the disease.

Both *APRT* and *LCAT* are localized to chromosome 16. While these two genes have not been previously implicated in COPD, this experiment showed novel insights into the metabolic dysregulation underlying this disease. APRT, a key mediator of purine salvage pathways (45), may influence cellular energy homeostasis through its regulation of AMP levels—a mechanism of particular relevance given the chronic oxidative stress and impaired energy metabolism characteristic of COPD. LCAT, though best characterized in cardiovascular cholesterol metabolism (52, 53), exhibits pulmonary-specific downregulation in this cohort, suggesting its potential role in handling and subsequent inflammatory amplification. Critically, GSEA revealed shared enrichment of both biomarkers in oxidative phosphorylation and ribosomal pathways, which aligned with established COPD hallmarks, such as mitochondrial dysfunction in airway epithelia and proteostasis imbalance in immune cells (13, 14). Then，immune infiltration analysis, regulatory networks and drug prediction and molecular docking was conducted for APRT and LCAT. These correlations provided initial insights into the potential interactions between LCAT, APRT, and specific immune cell subsets. APRT is involved in the purine rescue pathway and generates adenosine monophosphate (AMP) (45), which may affect the energy metabolism and proliferation of immune cells. LCAT is responsible for cholesterol esterification (53), which may affect the integrity of cell membranes and signal transduction, thereby regulating the activation and killing function of immune cells. Future functional studies could be performed to determine whether LCAT and APRT directly affect the proliferation, differentiation, or function of these immune cells.

Given the established roles of APRT in purine salvage pathways and LCAT in reverse cholesterol transport, future studies could prioritize developing targeted small-molecule therapeutics to modulate purine and cholesterol metabolic pathways, thereby intervening in COPD-associated metabolic reprogramming. Furthermore, the emerging evidence of their immune-metabolic crosstalk—particularly with CD56dim nature killer cells and alveolar macrophages—opens avenues for precision immune-metabolic therapeutic strategies, such as engineered metabolic checkpoint inhibitors or lipid metabolism-modulating biologics tailored to COPD endotypes. This dual targeting approach may synergistically restore metabolic-immune homeostasis while mitigating disease progression.

While current diagnostic guidelines establish pulmonary function testing as sufficient for COPD diagnosis, this study identified APRT and LCAT as possible key regulators of mitochondrial bioenergetics dysregulation in COPD pathogenesis, with preliminary validation in lung tissues. Although these genes currently lack utility as molecular biomarkers for early detection, they provide a solid theoretical foundation for understanding COPD-associated mitochondrial dysfunction and propose novel perspectives for early metabolic intervention strategies and therapeutic target discovery - particularly through their mechanistic links to emerging therapeutic paradigms such as immune-metabolic reprogramming and mitochondrial quality control in chronic airway diseases. Notably, current research on APRT has predominantly focused on the relationship between APRT deficiency and childhood renal dysfunction (49), while studies on LCAT have primarily centered on its cardioprotective effects in male populations with atherosclerosis (57). These findings regarding age- and sex-specific manifestations in prior studies underscore the necessity to prioritize investigating the relationships between age/gender and these biomarkers in future COPD studies.

However, this study has certain limitations: The clinical translation of these biomarkers requires validation in larger cohorts and confirmation through functional experiments. The limited sample size of this validation cohort represented a statistical power limitation in this study, particularly given the inherent biological heterogeneity of COPD populations. Pulmonary tissue specimens inherently require invasive surgical procedures such as lobectomy, particularly complicating sample collection in COPD patients due to their compromised pulmonary function and stricter surgical eligibility criteria. A multipronged validation strategy was outlined for future. First, expanding collaborations with thoracic surgery centers to prospectively collect a larger surgical cohort with balanced gender representation and broader age stratification COPD patients in four stages (mild, moderate, severe, and very severe) and a more comprehensive set of clinical characteristics. Second, complementary validation using less invasive longitudinal —including peripheral blood and research bronchoscopy-derived bronchoalveolar lavage fluid (BALF). Third, the expression patterns of APRT and LCAT within distinct cell types, such as epithelial cells, vascular endothelial cells, and stromal cells, could be analyzed and verified in future investigations.

In summary, leveraging datasets from the GEO database and employing integrative bioinformatics approaches, this study identified MM-associated biomarkers with potential therapeutic relevance in COPD. Comprehensive analyses elucidated their biological pathways, immune signatures, molecular regulatory networks (including miRNA-mRNA and TF-mRNA interactions), and pharmacological predictions. Preliminary validation was performed using clinical specimens. Despite limitations mentioned, the novel findings provide a foundational framework for future research. Ongoing investigations will focus on mechanistic exploration of these biomarkers to advance their therapeutic application in COPD management.

## Data Availability

The datasets presented in this study can be found in online repositories. The names of the repository/repositories and accession number(s) can be found in the article/Supplementary material.

## Footnotes

^1^ https://www.ncbi.nlm.nih.gov/geo/

^2^ http://www.string-db.org/

^3^ http://www.broadinstitute.org/msigdb

^4^ http://mirwalk.umm.uni-heidelberg.de/

^5^ https://ophid.utoronto.ca/mirDIP

^6^ http://bioinfo.life.hust.edu.cn/hTFtarget/

^7^ http://ctdbase.org/

^8^ https://www.wwpdb.org/

^9^ https://pubchem.ncbi.nlm.nih.gov/

^10^ http://cao.labshare.cn/cb-dock/
